# Criteria for Selecting the Optimal Method of Treating Hepatic Hemangiomas

**DOI:** 10.17691/stm2020.12.1.13

**Published:** 2020

**Authors:** A.V. Bazayev, A.R. Kokobelyan, D.S. Akulenko, A.N. Kudryavtseva, A.A. Malov

**Affiliations:** Associate Professor, Head of the Department of General, Operative Surgery and Topographic Anatomy, Privolzhsky Research Medical University, 10/1 Minin and Pozharsky Square, Nizhny Novgorod, 603005, Russia; Associate Professor, Department of General, Operative Surgery and Topographic Anatomy, Privolzhsky Research Medical University, 10/1 Minin and Pozharsky Square, Nizhny Novgorod, 603005, Russia; Student, Medical Faculty, Privolzhsky Research Medical University, 10/1 Minin and Pozharsky Square, Nizhny Novgorod, 603005, Russia; Student, Medical Faculty, Privolzhsky Research Medical University, 10/1 Minin and Pozharsky Square, Nizhny Novgorod, 603005, Russia; Associate Professor, Department of General, Operative Surgery and Topographic Anatomy, Privolzhsky Research Medical University, 10/1 Minin and Pozharsky Square, Nizhny Novgorod, 603005, Russia

**Keywords:** hepatic hemangioma, radiofrequency thermoablation, endovascular embolization, liver resection, minimally invasive interventions

## Abstract

**The aim of the study** was to develop criteria for optimal tactics of treating hepatic hemangiomas of various sizes and localization including endovascular, percutaneous puncture ablative, and open resection interventions.

**Materials and Methods:**

The results of treating 95 patients (65 women and 30 men aged 26–65 years) with hepatic hemangiomas have been analyzed. Tumor diagnosis was based on the data of echosonoscopy, MRI, multispiral computed tomography with intravenously injected contrasting medium, US dopplerography, and puncture biopsy. 78 patients were operated on, 63 of them underwent isolated surgery, whereas 15 patients were treated with a combination of methods. In 17 cases, the decision was made not to use operative treatment.

**Results:**

After open resection operations (n=34), complications in the form of bilomas were observed in 3 patients in the postoperative period, in 1 patient the tumor growth continued two months after the resection of liver segment IV. Sclerotherapy of hepatic hemangiomas with ethanol (n=13) resulted in the recovery of 10 patients, massive intravascular hemolysis has developed in one patient, two patients died. After radiofrequency thermoablation of hepatic hemangiomas less than 5 cm in diameter (n=4), recovery was achieved. Echosonoscopy showed the reduction of blood flow and absence of tumor growth in 12 patients after isolated endovascular embolization of the vessel nourishing hepatic hemangioma. The combined treatment according to the method developed by us resulted in clinical recovery of all 15 patients.

**Conclusion:**

Sclerotherapy of hepatic hemangiomas with ethanol, especially those being large in size, may cause unpredictable complications and individual pathological reactions with severe outcomes. Surgical treatment is not required if morphologically verified hepatic hemangiomas are less than 3 cm in diameter without evident clinical manifestations and growth. When the diameter of hepatic hemangiomas is in the range of 3–5 cm with a tendency to growth, radiofrequency thermoablation is preferred. Hemangiomas of the left liver lobe more than 5 cm in size should be treated by resection methods. Our combined method is designed to treat hemangiomas of the right liver lobe exceeding the size of 5 cm. If the right lobe tumor is more than 10 cm, it is advisable to make a decision in favor of open operation.

## Introduction

Hemangioma represents endothelial hyperplasia accounting for 84.6% of all benign hepatic tumors [1, 2]. The majority of them are cavernous and capillary. Solitary hemangiomas occur more commonly. Their size ranges between several millimeters to 40 cm [3]. Liver is supplied with blood from the common hepatic artery and portal vein. Normally, blood volume flowing through the system of the hepatic artery makes 15–30% of the liver bloodstream [4]. Although the tumor itself consists mainly of sinusoid and venous types of caverns, the hepatic artery is the primary source of blood supply to hemangioma [4]. Tumor growth is associated with the effect of some hormones: glucocorticoids and estrogens. This accounts for the more common occurrence of tumors in women [2, 5].

Patients usually complain of discomfort, heaviness in the right hypochondrium [6]. Sometimes dyspeptic syndrome is noted [7]. Some complaints may appear depending on the hemangioma localization and size [8]. Portal hypertension is possible in case of the portal vein compression, mechanical jaundice may occur if bile ducts are compressed [9, 10]. Damaged hepatic tissue with impairment of bile duct integrity and vascular structures in hepatic hemangioma as well as in other diseases may lead to hemobilia [11]. Thrombosis of hemangioma, thrombus infection and abscess formation are also possible. Sometimes thrombotic masses can be the source of pulmonary artery thromboembolism. One more complication in case of large hemangiomas is the Kasabach–Merritt syndrome (blood clotting disorder) which features thrombocytopenia with petechial hemorrhages on the skin. In multiple hepatic hemangiomatosis, the development of hepatomegaly, chronic hypoxia, compression of the liver parenchyma with the following cirrhosis and hepatocellular insufficiency is possible. When hemangioma pedicle (if present) is twisted, acute abdomen develops, while adhesion with the amentum or intestine loops may cause bowel obstruction. But the most severe complication is hemangioma rupture and, as a consequence, internal bleeding. Mortality rate in this case may reach 60–83% [9]. Hemangiomas are benign neoplasms but cases of their malignization have been reported [11]. The main methods used to diagnose hepatic hemangioma are as follows: US examination, multispiral computed tomography (MSCT), contrast-enhanced MRI, US dopplerography, contrast-enhanced US, and angiography. Morphological verification using percutaneous biopsy is also possible, though there is a risk of bleeding [10].

Indications to treatment of hemangiomas are considered when clinical symptoms are marked, there are complications (rupture with hemorrhage, hemobilia, thrombosis, central necrosis, compression of vessels and bile ducts), rapid hemangioma growth, the diagnosis is uncertain and malignant character of the neoplasm in the liver is impossible to exclude [6, 8].

The tactics of hemangioma treatment is not completely defined. It is difficult to suppose what is more reasonable: the risk of rupture or that of the operation. Some authors consider resections to be the only method of hemangioma treatment grounding such approach by the risk of complications [4, 6]. There are data on using enucleation of hemangioma located in the anterior liver segments [10]. At present, embolization of the afferent vessel is widely used in hemangioma management. Some authors believe this method to be most effective and minimally invasive [12]. Medical felt, particles of polyvinyl alcohol, metal coils, lipiodol, or helium particles may serve as embolizing agents [5, 13, 14]. Method of percutaneous puncture sclerotherapy has also found a wide application. The main idea of this method consists in the US-guided transcutaneous puncture of hemangioma with subsequent introduction of the sclerosing agent in the center of the tumor [15]. Injections of 96% ethyl alcohol have been established to cause formation of thrombi in the tumor vessels [4]. Some authors employ intraoperative sclerotherapy with the following liver resection to decrease blood loss [15]. One of the surgical methods of hemangioma treatment is cryosurgical intervention. Liver cryoresection and cryodestruction of separate small hemangiomas are performed in this kind of operation [16].

Presently, microwave ablation becomes more popular and is considered as a promising and minimally invasive technique for treatment of hepatic hemangiomas [9]. Method of US-guided radiofrequency thermoablation (RFTA) is also used in hemangiomas below 9.5 cm in size with good results, fibrosis, and 38–79% reduction of tumor volume [17]. Liver thermoablation using microwave energy has also been suggested [18]. However, the RFTA method has not gained wide application due to some technical problems. For example, the main reason limiting its application for treating hepatic hemangiomas is a significant loss of heat and a nonuniform tumor warming during the procedure due to its rich vascularization and rapid heat removal.

**The aim of the study** was to define optimal tactics for treatment of hepatic hemangiomas of various sizes and localization including endovascular, percutaneous puncture ablative, and open resection interventions.

## Materials and Methods

In the period from 2003 to 2018, 95 patients with hepatic hemangiomas were referred from the district medical settings to the Clinic of General, Operative Surgery and Topographic Anatomy of Privolzhsky Research Medical University on the basis of the N.A. Semashko Nizhny Novgorod Regional Clinical Hospital (Nizhny Novgorod, Russia). The main reasons of these referrals were clinical manifestations such as heaviness or pains in the epigastric or right hypochondriac regions, dynamically observed hemangioma growth, large sizes of hemangiomas revealed for the first time with the risk of complication development, suspicion of the malignant tumor character.

There were 65 women and 30 men aged 26–65 years among the admitted patients. The diagnosis was made based on the data of US examination, MRI and IV contrast-enhanced MSCT, US dopplerography, and US-guided puncture biopsy. Of 78 operated patients, 63 underwent isolated surgery, whereas 15 patients were treated with a combination of methods. In 17 cases, it was decided not to use operative treatment.

The study complied with the Declaration of Helsinki (2013) and was approved by the Ethical Committee of Privolzhsky Research Medical University. Written informed consent was obtained from every patient.

In 63 patients, the following operative interventions were performed singularly: hemihepatectomy in 15 patients, anatomical and atypical resection of liver segments with a tumor in 19, infiltration percutaneous and intraoperative (including laparoscopic) alcoholization of hemangiomas in 13, RFTA in 4, selective endovascular embolization of hepatic artery proper branches in 12.

The tumor size was the main criteria of selecting patients for each of these methods. Thus, hemangiomas with the diameter over 10 cm were not considered for ablative interventions due to a long duration of RFTA, probability of massive tumor necrosis, and associated complications.

In 13 patients, sclerotherapy of hepatic hemangiomas was conducted with sterile 96% ethanol at the early stages of the investigation before a wide implementation of RFTA and on hemangiomas not exceeding the size of 5 cm. The sclerosant was introduced to the depth of 2.5–3.0 cm under the obligatory visual (in intraoperative introduction) or radiological control. To realize the latter, rentgenocontrast substrate (Ultravist) was added to the sclerosant which made it possible to increase viscosity and assess the distribution.

Percutaneous puncture RFTA as an independent method was employed in 4 patients with the tumor size below 5 cm.

Isolated endovascular tumor arterioembolization was done on 12 patients without subsequent RFTA due to the use of embospheres which may melt during radiofrequency heating. The procedure was accompanied by complete obliteration of the tumor arterial bed.

15 patients underwent endovascular embolization in combination with the following percutaneous puncture RFTA. The follow-up examinations 1, 3, 6, and 12 months after the interventions included US, MRI, puncture biopsy with histological investigation. Recovery was judged by the absence of recurrence or persistent tumor growth during 1 year, and prevalence of the fibrous component according to the control biopsy.

To improve the effectiveness of RFTA in hepatic hemangiomas we suggested using preliminary rentgenendovascular selective embolization of the arteries feeding the tumor in order to reduce the heat loss caused by the blood flow to the effective values during the procedure. The preliminary artery embolization resulted in the decrease of blood flow in the tumor influencing the heat loss during RFTA. It was confirmed by angiography and US dopplerography data, and the efficacy of the conducted treatment later was verified by intravenous contrast-enhanced US examination and control puncture biopsy.

It was decided not to administer any treatment to 17 patients whose hemangiomas were within 3 cm in diameter without a tendency to rapid growth (increase less than 25% in diameter during annual dynamic follow-up) after puncture biopsy with histological tumor verification. Observation of this group of patients for 1–3 years did not reveal any clinically significant changes demanding treatment.

## Results

After open resection operations, clinical recovery was achieved in 31 patients, in 3 of them complications were in the postoperative period. After segmental resections of the right liver lobe, bilomas appeared in the operative area in 2 cases cured by minimally invasive methods (US-guided percutaneous punctures). Postoperative biloma occurred in 1 patient after right hemihepatectomy. There were no complications after hemihepatectomy of the left-sided tumors. Persistent tumor growth was noted in 1 patient after the resection of the fourth liver segment 2 months later, transcutaneous RFTA was performed.

Sclerotherapy of hemangiomas with ethanol was carried out on 13 patients, recovery was achieved in 10 of them. One patient had a complication in the form of massive intravascular hemolysis. In 2 cases, ethanol sclerotherapy resulted in fatal outcome due to total pancreonecrosis in 1 patient and necrosis of the left liver lobe with the development of hepatic insufficiency in the other.

Four patients with hemangiomas less than 5 cm in diameter were cured by the isolated RFTA method.

In all 12 patients treated by isolated endovascular embolization of the feeding vessel, the tumor growth was not noted during 1–3 years of follow-up (US examination, MRI), US dopplerography showed diminished blood flow, intravenous contrast-enhanced US (SonoVue) revealed absence or deceleration of contrast agent accumulation. Complications were not found.

Complete clinical recovery was observed in 15 patients after the combined treatment according to the method suggested by us. There were no complications after percutaneous puncture US-guided RFTA in combination with preliminary endovascular occlusion of the nourishing vessel. The results of the conducted treatment of patients with hepatic hemangiomas using different techniques are shown in the Table.

**Table:** Results of surgical treatment of hepatic hemangiomas

Operations	Number of patients	Recovery	Complications	Fatal outcome
Hemihepatectomy	15	14	1	0
Liver resection	19	17	2	0
Sclerotherapy with ethanol	13	10	3	2
Radiofrequency thermoablation	4	4	0	0
Endovascular arterial embolization	12	12	0	0
Combined method	15	15	0	0
Total	78	72	6	2

Case presentation.

**Figure 1:**
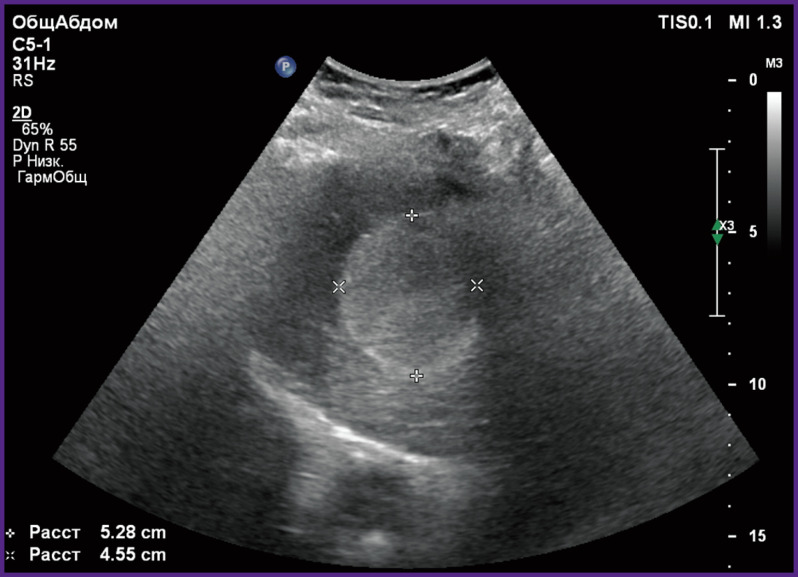
Patient L., 42 years old. Echosonogram of hepatic hemangioma

*A 42-year-old woman was treated for hemangioma of the right liver lobe. On admission, she complained of pains and heaviness in the right hypochondrium. US examination and MRI revealed hemangioma of the right liver lobe having 53 mm in diameter (Figure 1).*

**Figure 2:**
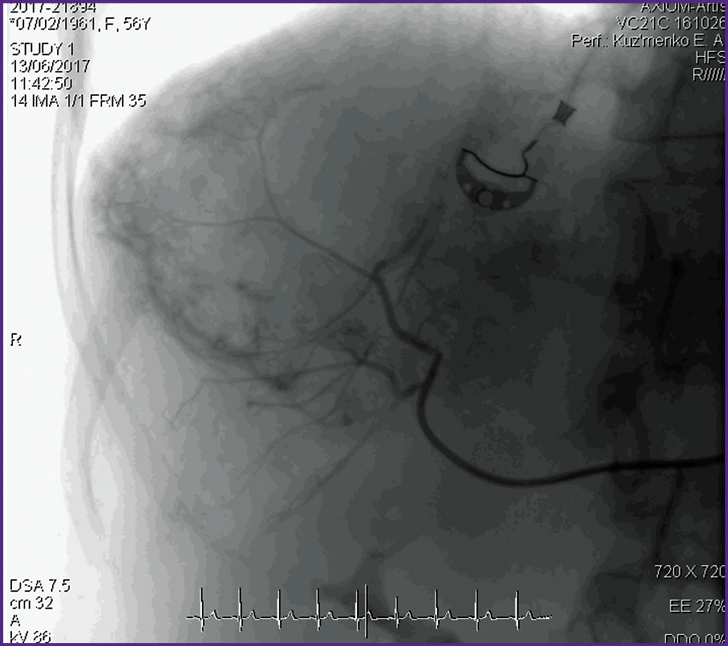
Angiography of hepatic hemangioma

*Contrast-enhanced angiography of hepatic arteries visualized pathological vascular network, intensive accumulation of the contrasting substance in the right lobe in the arterial, venous, and parenchymal phase, as well as shapeless units of the contrast matter (Figure 2).*

**Figure 3:**
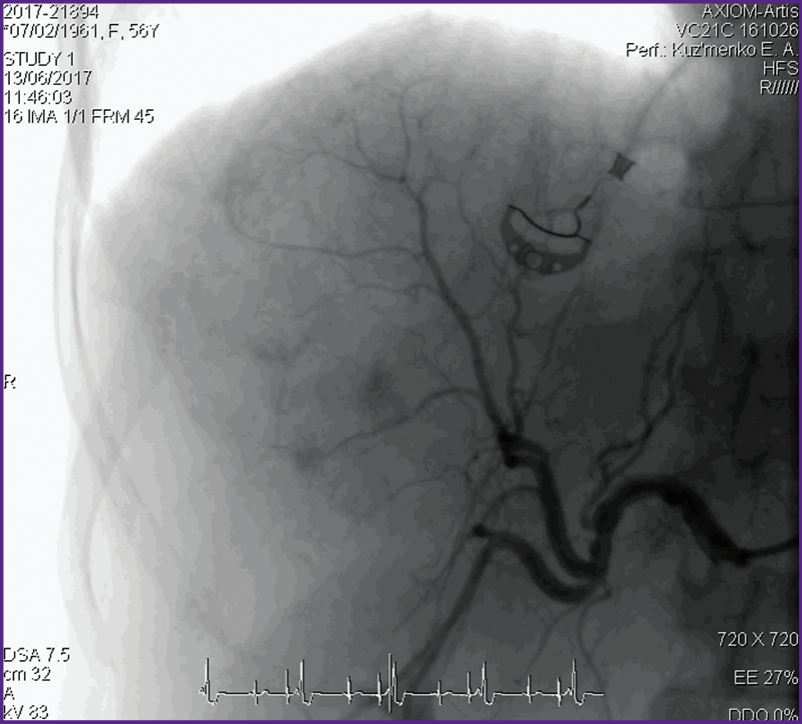
Preliminary endovascular embolization of the artery nourishing hemangioma

*After the additional examination, selective endovascular embolization of the artery nourishing the tumor: stagnation of the arterial blood flow in hemangioma has been achieved (Figure 3).*

**Figure 4:**
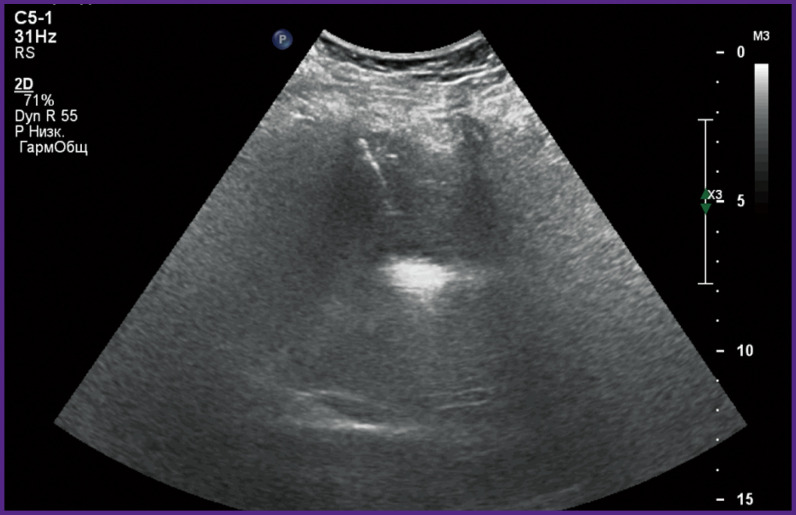
Tissue heating during RFTA of hepatic hemangioma

*On day 4, percutaneous puncture multifocal RFTA of the right liver lobe hemangioma (Figure 4) was performed.*

**Figure 5:**
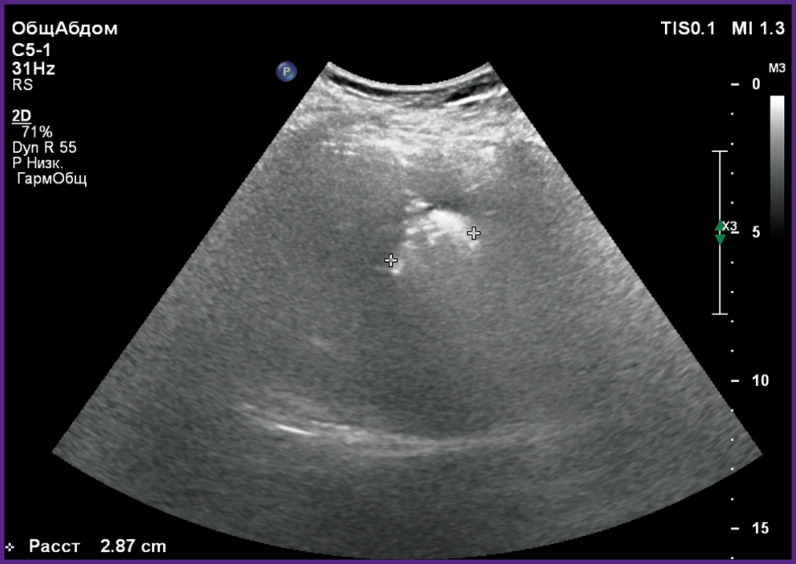
The result of combined treatment (preliminary endovascular embolization of the nourishing vessels and subsequent percutaneous puncture RFTA) of hepatic hemangioma

*The postoperative period was without complications. The control US examination showed that hemangioma shrank to 29 mm (Figure 5).*

*The control US examination with intravenously injected contrast agent revealed insignificant accumulation of the contrast matter in the arterial phase along the tumor periphery. Puncture biopsy performed 16 days later detected the elements of fibrous tissue. The patient was discharged recovered.*

## Conclusion

The analysis of the results of hepatic hemangioma surgical treatment using different methods allows us to formulate criteria which may guide the selection of the optimal tactics.

1. Sclerotherapy of hepatic hemangiomas, especially those having a large size, with ethanol may result in unpredictable complications and individual pathological reactions with severe outcomes.
2. Morphologically verified hemangiomas 3 cm or less in diameter without evident clinical manifestations and growth do not require any treatment.
3. Hepatic hemangiomas of 3–5 cm in diameter tending to grow should be treated by minimally invasive methods (percutaneous puncture RFTA is preferred).
4. Hemangiomas more than 5 cm in size located in the left liver lobe is better to treat with resection methods due to the specific anatomy of this region.
5. It is preferable to treat hemangiomas exceeding 5 cm in the right liver lobe with the suggested combined method which allows significant improvement of its efficacy. This method includes preliminary endovascular embolization of the nourishing vessels and subsequent percutaneous puncture RFTA of the tumor. The alternative to this method may be selective endovascular embolization of the tumor arterial network with embospheres without RFTA if there is a high risk of embolysate melting.
6. If the size of the right lobe tumor is more than 10 cm in diameter, it is advisable to make a decision in favor of the open operation.
7. The development of sclerofibrotic changes in the tumor tissue after minimally invasive interventions should be verified and monitored by the instrumental methods and puncture biopsy.
